# Associations Between Habitual School-Day Breakfast Consumption Frequency and Academic Performance in British Adolescents

**DOI:** 10.3389/fpubh.2019.00283

**Published:** 2019-11-20

**Authors:** Katie Adolphus, Clare L. Lawton, Louise Dye

**Affiliations:** Human Appetite Research Unit, School of Psychology, University of Leeds, Leeds, United Kingdom

**Keywords:** breakfast, academic performance, learning, adolescents, school performance, educational achievement

## Abstract

Studies indicate that breakfast positively affects learning in children. The present study aimed to examine associations between habitual school-day breakfast consumption frequency and academic performance, as measured by the General Certificate of Secondary Education (GCSE). The GCSE is a national academic qualification obtained by most British children during secondary education. Adolescents aged 16–18 years (*n* = 294; females: 77.2%) completed a retrospective 7-day food diary to report breakfast intake and a questionnaire to report GCSE grades. Breakfast was defined as any food or drink containing ≥5% of total energy expenditure (TEE) consumed up to 10:00 a.m. on school days. Habitual weekly school-day breakfast consumption frequency was categorized as rare (0–1 school days), occasional (2–3 school days), or frequent (4–5 school days). GCSE grades were aggregated into point scores and linear regression models were applied. Participants' GCSE grades in Mathematics and English were analyzed using ordinal logistic regression. Adolescents who rarely consumed breakfast on school days had a significantly lower capped point score (β = −0.13, *p* < 0.05) and mean point score (β = −0.14, *p* < 0.05) compared with frequent consumers. Low/middle socio-economic status (SES) adolescents who rarely consumed breakfast were significantly less likely to achieve higher Mathematics grades compared to low/middle SES adolescents who frequently consumed breakfast [adjusted cumulative odds ratio (OR): 0.35 95% confidence interval (CI): 0.17–0.72]. This cross-sectional study demonstrates that habitual school-day breakfast consumption amongst adolescents is a significant correlate of GCSE attainment. The results offer promising associative evidence which warrants further exploration in well controlled studies.

## Introduction

There is interest in the impact of dietary interventions for improving cognitive function in schoolchildren. Breakfast has the potential to improve children's cognitive function at school, which may benefit learning and academic performance. Numerous studies have examined the acute (same morning) and chronic effects of breakfast consumption on cognitive performance, as measured by objective cognitive function tasks. Systematic reviews have demonstrated that consuming breakfast relative to fasting has a positive effect on cognitive function measured within 4 h post-ingestion (1). However, the relationships are neither consistent nor clear-cut. The degree of improvement varies according to the nutritional status of the child (2, 3), as well as the timing and difficulty of the cognitive task employed (4–6).

Several studies have also considered the effects of breakfast on ecologically valid outcomes of academic performance in terms of school grades and achievement test scores (7–12). A systematic review of this evidence indicated that both habitual breakfast consumption and school breakfast programs are positively associated with academic performance in children and adolescents (13). Most support was found for Mathematics and arithmetic test performance (13). However, only 22 studies were included in the review, highlighting the paucity of research on ecologically valid academic outcomes (13). Furthermore, very few studies have examined a sample of adolescents from the United Kingdom (UK) and therefore, included measures of academic performance that are typically used in the British school system. Moreover, the majority of studies included in the review did not explicitly differentiate between school-day and weekend breakfast intake, despite the likely importance of school-day breakfast consumption in the relationship with academic attainment. More recently, cross-sectional studies have demonstrated that regular consumption of breakfast is associated with higher academic achievement (14, 15). However, a recent cross-sectional study failed to find a significant relationship between breakfast consumption and Cognitive Abilities Test (CAT) performance in a sample of British adolescents from low socio-economic status (SES) backgrounds (16).

Therefore, the aim of the present study was to examine associations between habitual school-day breakfast consumption frequency and academic performance in adolescents, as measured by the General Certificate of Secondary Education (GCSE). GCSE qualifications are gained following nationally administered courses taken by most 15–16 year olds during secondary education (high school) in England, Wales, and Northern Ireland. GCSE qualifications have a functional relevance to the pupil with both immediate and long term effects on eligibility to pursue further education, higher education, and employment (17–19).

## Methods

### Participants and Procedure

This was a cross-sectional study. Data collection was carried out between 2011 and 2012. Schools were recruited to take part in the study by coordinating with a senior teacher. The questionnaires were completed during a Research Open Day at the School of Psychology, University of Leeds. Adolescents participated with their class group and were chaperoned by their teachers. Three hundred and eleven adolescents [males: 70 (22.5%); females: 241 (77.5%)] aged 16–18 years were recruited to take part in this study. Participants were in full time education attending the final 2 years of secondary education (high school) in West Yorkshire, United Kingdom. Of the 311 participants recruited to take part, 17 (5.5%) returned incomplete food diaries or questionnaires or reported acute illness which may have influenced food diary data. Data from these 17 participants were, therefore, excluded from the statistical analysis. The final sample for analysis consisted of 294 participants [males: 67 (22.8%); females: 227 (77.2%)] aged 16–18 years. Ethical approval was obtained from the School of Psychology Research Ethics Committee at the University of Leeds, UK. The permission of the head teacher was received from each of the participating schools and parental consent was obtained prior to an adolescent's participation in the study. Participants gave oral consent on the day of data collection. Participant consent was indicated by the act of completing and returning the questionnaires.

### Measures

#### Demographic Characteristics

Participants completed a self-report written questionnaire to obtain demographic information including sex, age, ethnicity, and highest parent/guardian education level (used as a proxy for SES). Agreement between adolescents' and parents' reports of measures of SES are generally good (20), with the highest degree of agreement for reports of parental education compared with occupation or income (21). The height and weight of each participant was measured and recorded by researchers to determine body mass index (BMI) standard deviation scores (BMI SDS). BMI SDS were calculated using the LMS growth Microsoft Excel add-in which expresses BMI as a SDS based on British 1990 growth reference data (22, 23). Basal metabolic rate (BMR) was estimated using the pediatric height-weight equations proposed by Schofield (24). Total energy expenditure (TEE) was calculated as: BMR x Physical Activity Level (PAL), with PAL adjusted for energy needs for growth. The UK reference PAL value was used (PAL value 1.75) determined by the Scientific Advisory Committee of Nutrition (25).

#### Academic Performance

Participants' self-reported GCSE grades obtained during secondary education were used to measure academic performance. GCSE grades are awarded for each course subject, where pass grades include A^*^-G with U as ungraded/fail. To measure overall GCSE performance, aggregate point scores were created using the Department for Education GCSE point score system to transform individual subject grades into a continuous numerical point score (26). Participants' grades for each subject were transformed into corresponding point scores (e.g., A^*^ = 58, A = 52, B = 46 etc.) which were summed to produce a measure of overall GCSE performance. Three aggregate GCSE point scores were created: uncapped GCSE point score, capped GCSE point score, and mean GCSE point score. Uncapped GCSE point score is the total sum of point scores for all GCSE grades. Capped GCSE point scores is the total sum of the point scores from the highest eight GCSE grades only. Mean GCSE point score is the total uncapped point score divided by the total number of GCSE grades. Although there is some overlap, it was deemed necessary to perform separate analyses for each of these outcomes. Capped point score provides a measure of attainment that does not favor participants who have a greater number GCSE subject grades. Uncapped point score reflects both quality and the number of GCSE subject grades. To measure performance in Mathematics and English, participants' grades for each subject were used. Where participants reported more than one grade for English or Mathematics (e.g., English literature and English language), their best eligible result was used. Performance in Mathematics and English were of particular interest as these are compulsory GCSE subjects which are also included in performance indicators (e.g., 5 GCSE A^*^-C including Mathematics and English benchmark).

#### Habitual Breakfast Consumption

Participants completed a 7-day retrospective food diary using household measures to estimate weights of foods consumed. Participants were required to report all food and drink consumed for breakfast over a 7-day period which included the day of testing and the previous 6 days. No other meals or drinks were reported. To ensure consistency of reporting, participants were instructed that breakfast is defined as the first eating occasion involving a solid food or a drink that occurred after waking, up to and including 10:00 a.m. on school days/college days or 11:00 a.m. on weekend days (27). All food diaries were analyzed using WinDiets (Research Version 2010; Robert Gordon University, Aberdeen, UK).

A breakfast eating occasion was defined as any food or drink containing ≥5% of TEE consumed up to 10:00 a.m. on school days. The cut-off was adopted to represent a minimum amount of energy required for classification as breakfast. This cut-off equates to ~100 Kcal which represents the threshold of detection of food and beverage ingestion on appetite visual analog scales (28–30). Hence, the energy threshold for a breakfast eating occasion was at least capable of inducing changes in satiety and hunger. School-day (weekday) breakfast eating frequency was used to classify participants' habitual weekly school-day breakfast consumption as rare (0–1 school days), occasional (2–3 school days), or frequent (4–5 school days). The decision to use school-day breakfast intake frequency was *a priori* and based on several factors. Firstly, school-day and weekend breakfast habits often differ in terms of food type, time consumed, macronutrient, and micronutrient content (31–33). Secondly, school-day breakfast intake is temporally related to academic learning which may have immediate effects on the subsequent academic performance and cumulative effects on academic outcomes in the longer term. A 7-day retrospective food diary was used in this study because a further aim was to examine breakfast eating patterns in adolescents. These data are not reported in this paper.

### Statistical Analysis

Statistical analyses were performed using SPSS version 21 (SPSS, Inc. Chicago, USA) and the significance level (α-level) was set as *p* < 0.05. Hierarchical linear regression was conducted to examine the association between habitual school-day breakfast consumption and the three aggregate GCSE point scores variables: uncapped GCSE point score, capped GCSE point score (best 8 GCSE subjects taken), and mean GCSE point score. Model 1 was adjusted for ethnicity, age, sex, SES, and BMI SDS. Model 2 was adjusted for the covariates in model 1 in addition to interaction terms between socio-demographic variables (ethnicity, age, sex, SES, and BMI SDS) and habitual school-day breakfast consumption. Frequent habitual school-day breakfast consumption (4–5 school days) was the reference category in all analyses. Ordinal logistic regression (proportional odds model) was used to analyse the association between habitual school-day breakfast consumption and Mathematics and English GCSE grades. Potential differences in grades were expressed in cumulative adjusted odds ratios (ORs) and 95% confidence intervals (CIs). Ordinal logistic regression estimates cumulative ORs, modeling the probability of obtaining any higher GCSE grade across the entire grade variable. Parallel lines tests for habitual school-day breakfast consumption variables were non-significant confirming the assumption of proportional odds (34). Model 1 was adjusted for ethnicity, age, sex, SES, and BMI SDS. Model 2 was adjusted for the covariates in model 1 in addition to interaction terms between socio-demographic variables (ethnicity, age, sex, SES, and BMI SDS) and habitual school-day breakfast consumption.

## Results

### Participant Demographic Characteristics

Participant demographic characteristics are shown in Table 1. The sample consisted of 294 participants aged 16–18 years. There were significantly (*p* < 0.001) more females than males [males: 67 (22.8%); females: 227 (77.2%)]. Most participants were White British [241 (82.0%)] with fewer participants of any other ethnic background [53 (18.0%)]. Half of the participants were classified as high SES. The mean BMI SDS was 0.49 ±1.08. Mean (± SD) uncapped and capped GCSE point score was 524.10 ± 99.20 and 401.07 ± 37.95, respectively. The mean GCSE point score was 48.71 ± 3.93.

**Table 1:** Participant demographic characteristics.

**Demographic characteristics**	***n* (%)**
**Sex**	
Male	67 (22.8)
Female	227 (77.2)
**Ethnicity**	
White British	241 (82.0)
Other ethnic background	53 (18.0)
**SES**	
High SES	148 (50.3)
Low/middle SES	146 (49.7)
	**Mean (SD)**
**Height (cm)**	167.41 (8.89)
**Weight (kg)**	63.61 (13.02)
**BMI SDS**	0.49 (1.08)
**GCSE performance**	
Uncapped point score	524.10 (99.20)
Capped point score	401.07 (37.95)
Mean point score	48.71 (3.93)

### Habitual School-Day Breakfast Consumption

Approximately a third (28.6%) of participants rarely ate breakfast on school days. Over half the participants frequently ate breakfast on school days (53.1%). The remaining participants (18.4%) ate breakfast occasionally on schooldays.

### Associations With Academic Performance: Aggregated GCSE Performance

Table 2 details the results of the hierarchical multiple regression for uncapped and capped GCSE point score, and mean GCSE point score. Habitual school-day breakfast consumption frequency was not significantly associated with uncapped GCSE point scores. For capped GCSE point score, model 1 indicated that scores were 0.14 standard deviations lower in adolescents who rarely eat breakfast on school days compared with those who frequently consume breakfast (standardized beta coefficient [β] = −0.14, *p* < 0.05). After including interaction terms in model 2 this significant relationship remained (β = −0.13, *p* < 0.05). Converted to point scores, the fully adjusted unstandardized beta coefficient (B) indicated that capped GCSE point scores were on average 10.25 points lower in adolescents who rarely eat breakfast on school days compared to those who frequently eat breakfast (B = −10.25, 95% CI = −19.16 to −1.34; Table 2, model 2).

**Table 2:** Linear regression analyses of the association between habitual school-day breakfast consumption and aggregated GCSE performance.

**Model**	**Explanatory variables**	**Uncapped point score**			**Capped point score**			**Mean point score**		
		**B**	**SE B**	**β**	**B**	**SE B**	**β**	**B**	**SE B**	**β**
**1^a^**	**Habitual school-day breakfast**									
	Frequent (reference)									
	Occasional	−20.79	14.01	−0.09	−0.37	5.08	0.00	0.21	0.55	0.02
	Rare	−16.29	12.26	−0.08	−11.30	4.43	−0.14^*^	−1.25	0.48	−0.14^**^
**2^b^**	**Habitual school-day breakfast**									
	Frequent (reference)									
	Occasional	−25.01	14.37	−0.11	−1.54	5.24	−0.02	0.09	0.57	0.01
	Rare	−12.44	12.42	−0.06	−10.25	4.53	−0.13^*^	−1.20	0.49	−0.14^*^
	**Interaction terms**									
	Ethnicity ^*^ Occasional breakfast	5.54	36.24	0.01	1.72	13.23	0.01	0.54	1.45	0.02
	Ethnicity ^*^ Rare breakfast	−12.68	32.14	−0.02	7.47	11.73	0.04	1.02	1.28	0.04
	SES ^*^ Occasional breakfast	−22.76	28.83	−0.05	−1.27	10.52	−0.01	0.39	1.15	0.02
	SES ^*^ Rare breakfast	12.86	24.41	0.03	7.00	8.90	0.04	0.79	0.97	0.05
	Sex ^*^ Occasional breakfast	−2.16	38.48	0.00	7.39	14.03	0.03	1.79	1.53	0.07
	Sex ^*^ Rare breakfast	−54.96	29.37	−0.11	−8.27	10.69	−0.04	−0.22	1.17	−0.01
	Age ^*^ Occasional breakfast	−0.21	18.97	0.00	3.09	6.92	0.03	0.37	0.76	0.03
	Age ^*^ Rare breakfast	27.92	16.11	0.10	6.22	5.87	0.06	0.19	0.64	0.02
	BMI SDS ^*^ Occasional breakfast	2.18	13.14	0.01	0.55	4.79	0.01	0.02	0.52	0.00
	BMI SDS ^*^ Rare breakfast	−13.25	11.20	−0.07	−5.66	4.07	−0.08	−0.43	0.44	−0.06

a *Adjusted for ethnicity, SES, sex, age, and BMI SDS*.

b *Adjusted for ethnicity, SES, sex, age, BMI SDS, and interaction terms*.

* *p <0.05*,

** p <0.01, ^***^p <0.001.

Similar results were found for mean GCSE point score. The adjusted β coefficients (Table 2, model 1) indicated that mean GCSE point score was 0.14 standard deviations lower in adolescents who rarely eat breakfast on school days compared with those who frequently eat breakfast (β = −0.14, *p* < 0.01) which remained significant following adjustment for interaction terms in model 2 (β = −0.14, *p* < 0.05). In terms of point scores, mean GCSE point scores were on average 1.20 points lower in adolescents who rarely eat breakfast on school days compared to those who frequently eat breakfast, controlling for covariates (B = −1.20, 95% CI = −2.17 to −0.23; Table 2, model 2).

Occasional school-day breakfast consumption was not significantly related to all aggregate GCSE point scores in all models. Additionally, all interaction terms were non-significant in all models.

### Associations With Academic Performance: Mathematics and English Grades

Table 3 details the results of the ordinal logistic regression for GCSE Mathematics and English grades. For English grades, model 1 indicated that adolescents who rarely eat breakfast on school days had significantly lower cumulative odds of achieving higher grades than adolescents who frequently eat breakfast (adjusted OR = 0.57, 95% CI = 0.35–0.95, *p* < 0.05). In model 2 with interaction terms, neither occasional nor rare school-day breakfast consumption was significantly associated with English grades.

**Table 3:** Ordinal logistic regression (proportional odds model) for GCSE English and Mathematics grades.

**Model**	**Explanatory variables**	**English**	**Mathematics**
		**Cumulative OR^a^ (95% CI)**	**Cumulative OR^a^ (95% CI)**
**1^b^**	**Habitual school-day breakfast**		
	Frequent (reference)	1.00	1.00
	Occasional	0.73 (0.41–1.31)	1.04 (0.58–1.84)
	Rare	0.57^*^(0.35–0.95)	0.63 (0.39–1.03)
**2^c^**	**Habitual school-day breakfast**		
	Frequent (reference)	1.00	1.00
	Occasional	0.57 (0.11–2.91)	0.74 (0.15–3.72)
	Rare	0.49 (0.14–1.69)	0.26^*^(0.07–0.88)
	**Interaction terms**		
	Ethnicity ^*^ Occasional breakfast	1.34 (0.29–6.15)	1.10 (0.24–5.01)
	Ethnicity ^*^ Rare breakfast	2.11 (0.55–8.16)	1.47 (0.39–5.60)
	SES ^*^ Occasional breakfast	1.02 (0.31–3.39)	1.24 (0.38–4.06)
	SES ^*^ Rare breakfast	1.61 (0.57–4.54)	3.98^**^(1.42–11.16)
	Sex ^*^ Occasional breakfast	1.25 (0.25–6.15)	1.26 (0.26–6.19)
	Sex ^*^ Rare breakfast	0.72 (0.22–2.35)	1.09 (0.34–3.48)
	Age ^*^ Occasional breakfast	0.65 (0.29–1.45)	0.93 (0.42–2.06)
	Age ^*^ Rare breakfast	0.72 (0.38–1.37)	0.69 (0.37–1.30)
	BMI SDS ^*^ Occasional breakfast	1.14 (0.65–1.98)	0.85 (0.49–1.47)
	BMI SDS ^*^ Rare breakfast	1.06 (0.66–1.68)	1.45 (0.91–2.29)

a *Shown are cumulative ORs for higher grades*.

b *Adjusted for ethnicity, SES, sex, age, and BMI SDS*.

c *Adjusted for ethnicity, SES, sex, age, BMI SDS, and interaction terms*.

* *p <0.05*,

** p <0.01, ^***^p <0.001.

For Mathematics grades, habitual school-day breakfast consumption was not significantly associated with grades in model 1. However, in model 2 including interaction terms, there was a significant association between rare school-day breakfast consumption and Mathematics grades. The adjusted OR indicated that rare school-day breakfast consumption was associated with lower cumulative odds for higher Mathematics grades compared to frequent school-day breakfast consumption (adjusted OR = 0.26, 95% CI = 0.07–0.88, *p* < 0.05). A significant interaction was observed between SES and rare school-day breakfast consumption. To explore this interaction, the analysis was stratified by SES group. This indicated that only those adolescents from low/middle SES backgrounds who rarely eat breakfast on school days had significantly lower cumulative odds for higher Mathematics grades (adjusted OR = 0.35 95% CI = 0.17–0.72, *p* < 0.01) compared to those who frequently eat breakfast. There were no significant associations between habitual school-day breakfast consumption and Mathematics grades in adolescents from higher SES backgrounds. All other interaction terms were non-significant. Occasional school-day breakfast consumption was non-significant in all models for both Mathematics and English grades.

## Discussion

### Overview of the Findings

The present study examined the relationship between habitual school-day breakfast consumption frequency and GCSE attainment, a national academic qualification obtained by most British school adolescents during secondary education. This study examined a sample of 16–18 year old UK adolescents. Compared to national GCSE performance at the time of data collection, this sample was a relatively high achieving sample of adolescents (35).

Rare school-day breakfast consumption was negatively associated with measures of aggregated GCSE performance after controlling for confounders. Capped GCSE point scores were on average 10 points lower and mean GCSE point scores were on average 1 point lower in adolescents who rarely eat breakfast on school days compared to those who frequently eat breakfast. The magnitude of the effects suggests meaningful differences in GCSE grades (Department for Education point score scale increases by 6 points for each grade increase). The findings are consistent with previous research demonstrating that regular breakfast consumption is positively associated with academic performance in children and adolescents, outlined in our previous systematic review (13). The results are also in accordance with a recently published study which reported that regular breakfast consumption (7 days per week) was strongly associated with increased odds of high self-reported academic achievement in Norwegian adolescents aged 15–17 years, particularly in girls (14).

Aggregated GCSE attainment decreased with lower frequencies of school-day breakfast consumption. However, a significant association was apparent only when comparing rare school-day breakfast consumption (on 0–1 school days/week) to frequent (on 4–5 school days/week). This suggests that significant effects on GCSE performance are more evident at the extremes of breakfast consumption.

Uncapped GCSE point scores were not associated with breakfast consumption on school days. However, capped point score (best 8) and mean GCSE point score were negatively associated with breakfast skipping (0–1 days per weekday) on school days. These measures are not indicative of the number of GCSE grades, but reflect the quality of the results obtained. These findings suggest that habitual school-day breakfast consumption has greater effects on the quality of the grades rather than the number of GCSE grades achieved. It is unlikely that habitual school-day breakfast consumption has a strong effect on the number of GCSE grades achieved in different subjects. This may be because the number of GCSE grades achieved is more influenced by school factors, rather than habitual breakfast consumption. School pupils have a set choice regarding which subjects are studied at GCSE level. There are generally three compulsory subjects: English, Mathematics, and Science. All other subjects taken at GCSE are optional. Optional GCSE subjects offered vary between schools. For example, some GCSE subjects may not be available, particularly a language subject like German, French, or Spanish. Furthermore, each school determines the number of GCSEs its pupils can take, which could be as many as 12 or as few as 7.

The association between habitual school-day breakfast consumption frequency and aggregated GCSE performance was consistent across SES, ethnicity, sex, age, and BMI SDS. No interaction terms were significant and the inclusion of interaction terms into the regression models did not improve the models. Collectively, this suggests that in this sample of adolescents, there is no evidence of systematic variation in the effects of habitual school-day breakfast consumption frequency on GCSE attainment across socio-demographic groups. However, this finding should be interpreted with caution as the sample was not sufficiently diverse to detect reliable interactions between socio-demographic variables and breakfast consumption patterns.

There were no clear subject differences for Mathematics and English. Rarely eating breakfast on school days was associated with poorer grades in both subjects, but this was slightly attenuated following adjustment for covariates and interaction terms. However, the association between habitual school-day breakfast consumption frequency and GCSE attainment appeared stronger for Mathematics grades. Rarely consuming breakfast on school days was significantly associated with poorer Mathematics attainment, which is largely consistent with previous work (13). Further, SES background modifies the association between habitual breakfast consumption and GCSE Mathematics attainment. Examination of interaction effects indicated that rare school-day breakfast consumption was significantly associated with poorer Mathematics attainment in low/middle SES adolescents only. This suggests that the negative effects of skipping breakfast on school days are more apparent for Mathematics attainment, but only for adolescents from low/middle SES backgrounds. In high SES adolescents, overall quality of the diet (including evening intake) may be higher than in low SES adolescents which may offset any detrimental effects of skipping breakfast. For example, SES discrepancies in dietary intake are observed for intake of fruit, vegetables, fish, whole grains, fiber rich foods, and high fat food (36–39). The associations between SES and diet may exist because children and adolescents from families of higher SES may have more knowledge about healthy dietary choices and more income for healthier food. Previous work has also shown positive effects of breakfast consumption on academic performance in low SES groups (40, 41). However, no study has directly compared the effects of breakfast on attainment in children and adolescents of differing SES and some studies suggest that effects are evident in all SES groups (13).

### Strengths and Limitations

The study extends previous work to include British adolescents and the important GCSE phase of secondary schooling. GCSE attainment was assessed by typical performance indicators used within the education system. The focus on school-day breakfast consumption in this relationship is novel and renders the study findings particularly relevant for informing school-based interventions. However, the public health relevance of, and implications of the findings, are restricted at present due to the observational design of the study. Although trials are needed to establish whether altering the breakfast habits of adolescents can alter their academic attainment, these findings are encouraging.

A number of methodological limitations should be considered when interpreting the results. The cross-sectional design precludes causal inferences. Furthermore, the observed breakfast eating habits may not reflect habits at the time of examination and preparation for GCSE qualifications, nor learning prior to this period. However, breakfast eating habits formed during adolescence tend to stay stable. Regular breakfast consumption during adolescence significantly predicts regular breakfast consumption during young adulthood (42). It is also important to allow for the potential of unmeasured and residual confounding. It is probable that there is some residual confounding in the results with respect to SES. The study relied on adolescents' reports of parental SES (measured as highest parent/guardian education level) which may have introduced some measurement error in the estimation of parental SES. Breakfast consumption can coexist with other lifestyle factors highlighting the possibility of unmeasured confounding. Employing a retrospective 7-day food diary has limitations due to the reliance on participants' memory (43). The use of a self-report measure of academic performance may have introduced recall error and encouraged socially desirable responses. However, evidence suggests that self-reported academic performance correlates highly with actual academic performance (44). The recruitment method caused an unintended recruitment bias of a homogenous sample of high achieving adolescents, in which male adolescents, lower SES and ethnic minority groups were underrepresented. Finally, breakfast quality was not considered in the analysis and therefore conclusions regarding what aspects of breakfast are correlates of academic performance cannot be drawn.

## Conclusions

In conclusion, the findings suggest that regular breakfast consumption on school days is a significant correlate of adolescents' academic performance at age 16–18 years after controlling for covariates. Breakfast skipping was found to be negatively associated with GCSE performance, a finding supported by previous observational research. However, the cross-sectional design only confirms the coexistence of breakfast skipping and lower academic performance in the adolescents studied. Given the multiplicity of interacting factors influencing academic attainment in adolescents, teasing out the independent effects of breakfast is a considerable challenge which requires careful examination in further studies.

## Data Availability Statement

The raw data supporting the conclusions of this manuscript will be made available by the authors, without undue reservation, to any qualified researcher.

## Ethics Statement

The studies involving human participants were reviewed and approved by School of Psychology Research Ethics Committee at the University of Leeds, UK. Written informed consent from the participants' legal guardian/next of kin was not required to participate in this study in accordance with the national legislation and the institutional requirements.

## Author Contributions

KA conceived and designed the study, collected, analyzed and interpreted the data, and drafted and wrote the manuscript. LD and CL contributed to the design of the study, interpretation of the data, and revised the manuscript critically. All authors read and approved the final manuscript.

### Conflict of Interest

The authors declare that the research was conducted in the absence of any commercial or financial relationships that could be construed as a potential conflict of interest.
